# Physical activity levels six months after a randomised controlled physical activity intervention for Pakistani immigrant men living in Norway

**DOI:** 10.1186/1479-5868-9-47

**Published:** 2012-04-26

**Authors:** Eivind Andersen, Nicola W Burton, Sigmund A Anderssen

**Affiliations:** 1 Department of Sport Medicine, Norwegian School of Sport Sciences, Ullevaal Stadium, Box 4014, 0806, Oslo, Norway; 2 School of Human Movement Studies, The University of Queensland, Brisbane, Australia

**Keywords:** Physical activity, Immigrants, Psychosocial mediators, Long term follow-up

## Abstract

**Background:**

To our knowledge, no studies have aimed at improving the PA level in south Asian immigrant men residing in Western countries, and few studies have considered the relevance of SCT constructs to the PA behaviour of this group in the long term. The observed low physical activity (PA) level among south Asian immigrants in Western countries may partly explain the high prevalence of cardiovascular diseases (CVD) and type 2 diabetes (T2D) in this group. We have shown previously in a randomised controlled trial, the Physical Activity and Minority Health study (PAMH) that a social cognitive based intervention can beneficially influence PA level and subsequently reduce waist circumference and insulin resistance in the short-term. In an extended follow-up of the PAMH study: we aimed 1) to determine if the intervention produced long-term positive effects on PA level six months after intervention (follow-up 2 (FU2)), and 2) to identify the social cognitive mediators of any intervention effects.

**Methods:**

Physically inactive Pakistani immigrant men (n = 150) who were free of CVD and T2D were randomly assigned to a five months PA intervention or a control group. Six months after the intervention ended, we telephoned all those who attended FU1 and invited them for a second follow-up test (FU2) (n = 133). PA was measured using ActiGraph accelerometers. Statistical differences between groups were determined by use of ANCOVA.

**Results:**

Significant differences (baseline to FU2) between the groups were found for all PA variables (e.g., total PA level, sedentary time, PA intensity). Support from family and outcome expectancies increased more in the intervention group compared with the control group. Self-efficacy did not differ significantly between groups.

**Conclusions:**

Our results show that a multi component PA programme can increase PA over the short and long term in a group of immigrant Pakistani men. However, we could not identify the factors that mediated these changes in PA.

**Protocol ID:**

07112001326, **NCT ID:** NCT00539903

## Introduction

Lack of moderate- to vigorous- -intensity physical activity (MVPA) is associated with a higher all-cause mortality [1] and increased risk of developing coronary heart disease [2], type 2 diabetes (T2D) [3], and the metabolic syndrome [4]. A physically active lifestyle seems to be protective [5-11]. For example, in the Finnish Diabetes Prevention Study, participants who walked for an average of 2.5 h∙week^-1^ were 63% to 69% less likely to develop T2D than those who walked < 1 h∙week^-1^[12]. A follow-up study of the participants in Finnish Diabetes Prevention Study showed that the relatively brief but resource-demanding intervention reduced the incidence of T2D (relative risk reduction of 36%) many years after the intervention programme [13].

Adherence to a physically active lifestyle over the long term is essential to derive sustainable health effects. However, developing interventions where the physical activity (PA) behaviour is maintained over the long term has proven challenging. Fewer than half of those who initiate any type of PA regimen will not continue the behaviour [14], and when interventionists and the incentives they provide are no longer available, PA tends to decline with time [14].

Interventions may be more successful at inducing sustained behaviour change if they involve strategies of behaviour modification [14]. Social cognitive theory (SCT) [15] is acknowledged as one of the leading behaviour change theories to explain and predict PA in the general population [16,17] and in those with T2D [18]. The central tenet of SCT is self-efficacy (i.e., confidence to perform the behaviour), which has been consistently and positively associated with PA [19]. Another major SCT construct is outcome expectancies (i.e., the person must value the outcomes that he/she believes will occur and that these positive outcomes outweigh any negative outcomes that might also be experienced). Other SCT constructs include the physical environment, the social environment, behavioural capability, and personal regulation. PA interventions targeting these constructs may use strategies such as providing PA opportunities, social support, and skills training; identifying PA outcomes with functional meaning; and observational learning, rewards and incentives, goal setting, problem solving, and self-reward [20].

The Physical Activity and Minority Health (PAMH) study was a five month SCT based PA programme for sedentary Pakistani immigrant men living in Norway. The low levels of PA among south Asian immigrants (which includes those from Pakistan, India, Sri Lanka, Nepal, and Bangladesh) in Western countries [21-28] are likely to contribute to the high prevalence of T2D and CVD [21,22,29-36]. To our knowledge, only a few interventions have aimed at improving the PA level in south Asian immigrant men residing in Western countries, but none of these have reported on the long-term effects of the intervention [37]. In addition only one of these had a randomised controlled design [38]. This latter study was however, not designed to test the effect of a behaviour change program on the PA behaviour, but rather to test the effect of a structured exercise program over 12 weeks on insulin sensitivity. Also, few interventions have considered the relevance of SCT constructs to the PA behaviour of this group. As reported previously, a randomised controlled trial (RCT) of the intervention found positive short-term improvements (immediately after the intervention; follow-up 1 (FU1)) in PA level and accompanying beneficial changes in insulin sensitivity and waist circumference ([39] in press). The aims of the current study were: 1) to determine if the intervention produced long-term (six months after the intervention; follow-up 2 (FU2)) positive effects on PA level, and 2) to identify the social cognitive mediators of any intervention effects.

## Methods

The design, the intervention programme and methods of the PAMH study have been described in detail previously ([39] in press), a short description is given below. The study protocol was approved by the Regional Committee for Medical Research Ethics (ref. no. S-07300b) and the Norwegian Social Science Data Services (ref. no. 17212/2/KS). All participants gave written informed consent.

### Participants

**Figure 1:**
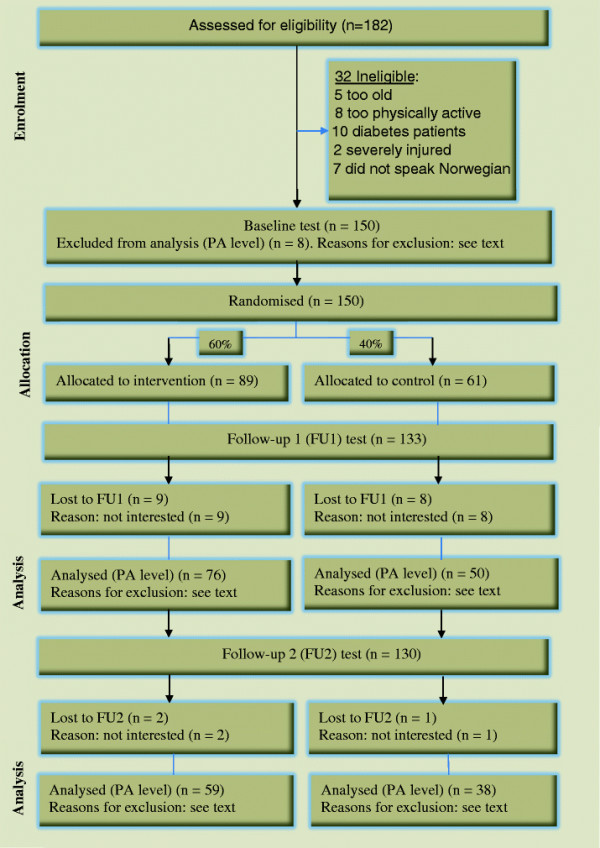
Flow of participants through the trial. FU1; follow up 1, FU2; follow-up 2.

For the original RCT, 150 participants, recruited through mosques and Muslim festivals, were randomised to either an intervention group or a control group at the baseline visit using a computer-generated list of random numbers. Pakistani (either born in Pakistan or parents born in Pakistan) immigrant men living in Oslo, Norway who were aged 25 to 60 years and who were not too physically active (i.e., they were excluded if they were exercising more than twice a week at a moderate or higher intensity for ≥ 30 min or cycling or walking to work most days of the week) were eligible for inclusion. Participants were not eligible if they had diabetes, did not speak Norwegian or had a severe injury. Seventeen participants were lost to FU1. Six months after the intervention ended, we telephoned all those who had attended FU1 and invited them to the FU2 (n = 133). Three participants declined, leaving 130 participants in the FU2 study. A scheme of the flow of participants through the trial is presented in the Figure 1.

### Intervention programme

Using the results from two focus groups with representatives from the male Pakistani immigrant group (n = 20) ([39] in press) and numerous studies supporting the use of SCT constructs to change PA behaviour [20], we targeted three primary SCT key concepts to promote PA change: self- efficacy (i.e., confidence to perform PA), social environment (i.e., social support for PA from family and friends, physically active role models), and outcome expectancies (i.e., expected benefits and costs of performing PA). The secondary SCT components targeted included the physical environment (opportunities to perform PA) and behavioural capability (knowledge and skill). The programme included structured group exercise sessions led by an exercise physiologist twice a week, two group lectures, one individual counselling session, written material and a phone call. Table 1 provides an overview of how these strategies were linked to SCT constructs. The intervention programme lasted five months. The control group received their baseline results about two weeks after the testing, and was offered organised exercise (once a week for four months), one group lecture and written material after the end of the intervention.

**Table 1:** Overview of the intervention components, attendance rates, behaviour change strategies and targeted social cognitive constructs

**Intervention component**	**Dose**	**Description**	**Behaviour change strategy**	**Targeted construct**
Structured group exercise	60 min twice a week	Participants could choose to attend one out of five different exercise facilities in Oslo. The different exercise groups were led by an exercise physiologist. The exercise training programme was designed as a low threshold activity. The sessions had the following structure: a 15 min warm-up with easy and fun games, 40 min of floor ball and/or football plus some strength exercises and a 5 min cool down. Seven participants did not attend any of the sessions (one trained by himself and six were not motivated) and two were injured at the first exercise session. The mean attendance was 60% (range: 11% to 100%).	-Provide opportunities for PA	-Environment
			-Increase social support for PA	-Expectancies
			-Promote mastery learning through skill training	-Self-efficacy
			-Improve knowledge and skill to perform PA	
			-Promote positive outcomes of PA	
			-Provide credible role models for PA	
Group lectures	2x2h	The lectures were conducted at the Norwegian School of Sports Sciences. The project leader led the classes. Major topics were:	-Improve knowledge of PA options, including non-vigorous PA	-Social support
		-What is PA?	-Improve knowledge on how to incorporate PA into the daily routine	-Expectancies
		-PA and health link; short- and long term effects	-Enhance PA expectancies	-Self-efficacy
		-The harms of physical inactivity	-Improve goal setting for PA	
		-PA recommendations and how to achieve these	-Improve problem solving of PA barriers	
		-Activity examples	-Improve social support for PA	
		-Setting small goals		
		-Identifying and reducing perceived barriers		
		-Making a PA plan		
		-Seeking social support		
		-Self reward		
		Both attendees (90%) and non-attendees received written summaries of the lecturers.		
Individual counselling sessions	1 h	The counselling was based on the concept that all advice must match the participants’ experience of PA and degree of motivation. Together with the participant, the primary goal was to find activities that could be implemented in a usual week, with the sum of these activities enabling them to reach the PA recommendations. After discussing activity options, the participants set the goals they wanted to achieve over the five-month period. Finally, we discussed barriers by asking “What do you think can stop you from carrying out this activity plan?”, and the possible barriers, and solutions to them were discussed and written down. All participants completed this part of the intervention.	-Identify opportunities for PA	-Social support
			-Improve knowledge and skill to perform PA	-Self-efficacy
			-Enhance goal setting for PA	-Expectancies
			-Promote mastery for PA	
			-Identify and problem solve barriers to PA	
Phone call	5-15 min	Three to five weeks before the first follow-up test, intervention participants in the intervention group were telephoned to discuss the activity plan, to make changes if necessary, and to encourage further efforts. All participants were reached within three attempts.	-Provide feedback on PA behaviour	-Social support
			-Reinforce problem solving for PA	-Self-efficacy
			-Provide encouragement and help	

### Measurements

The 130 participants who agreed to participate in the FU2 study were sent a package by mail containing a pre-programmed accelerometer and information on how and when to use it, a questionnaire and a prepaid envelope for return.

### Accelerometer recordings

Free-living PA was assessed using the ActiGraph accelerometers (ActiGraph, LLC, Pensacola, FL, USA). An accelerometer is an instrument that continuously measures acceleration, and the raw data from this instrument are called “counts”, which represent the sum of acceleration in a given time period. The activity monitor model 7164 was used at baseline and FU1, and the GT1M model at the FU2 test. The different types of Actigraph accelerometers have been shown to produce the same results during walking and running [40].

The primary outcome variable from the accelerometer data was the average counts per minute per day (CPM) (an indicator of the total PA level) throughout the seven-day measurement period. The secondary outcomes were the minutes spent sedentary behaviour, and in light-, moderate-, vigorous- and very vigorous-intensity PA using the following cut point (all in CPM): sedentary behaviour ≤ 100 CPM [41], light intensity PA, 101 to 1951 [42]; moderate intensity PA, 1952 to 5724; vigorous intensity PA, 5725 to 9497; and very vigorous intensity PA > 9497 [43]. These cut points are used widely and correlate with maximal oxygen uptake (r = 0.88) [43].

The participants were instructed to wear the accelerometer on the right hip during all waking hours, except while swimming and bathing, for seven days. The accelerometers were programmed to start recording at 6 am the day after the participants received their accelerometer. The epoch length (sample interval) was set to 1 min. In the analysis of accelerometer data, epoch periods with a value of zero for 60 min (with allowance for two exceptions above zero) or longer were interpreted as “accelerometer not worn” and removed from the analyses [42,44]. PA data were used if the participant had accumulated a minimum of eight hours of activity data per day for at least two days, regardless of the type of day (workday or weekends). CPM did not differ between those who wore the monitor for two days (baseline: n = 7, FU1: n = 3) and those who wore the monitor for three days or more. Subsequently, data from those participants who had worn the monitor for two days were also included [45]. Accelerometer data were processed and analysed using the SAS-based (version 9) (SAS Institute Inc. Cary, NC, USA) program CSA-Analyser (http://csa.svenssonsport.dk).

One hundred and forty-two participants (95%) had valid recordings at the baseline test: four lost their monitor and four had fewer than two valid days of recordings. At the FU1 test, 126 participants (84%) had valid recordings: 17 did not attend FU1, five had fewer than two days of recordings, and two did not return their accelerometer. Of the 130 participants at the FU2 test, we had valid accelerometer recordings on 97 (65%) of the original baseline sample: 12 participants sent the monitor back without having used it, six had fewer than two days of valid recordings and 15 did not return the monitor (Figure 1).

The participants wore the monitor for an average of 6.3 ± 1.8 days (mean ± standard deviation (SD)) at baseline, 6.1 ± 1.5 days at FU1, and 5.6 ± 1.6 days at FU2. The mean (± SD) wearing time was 13.5 ± 1.5 h∙day^-1^ at baseline, 13.6 ± 1.6 h∙day^-1^ at FU1 and 13.3 ± 1.9 h∙day^-1^ at FU2.

### Social cognitive variables

The following potential mediators of change in PA were measured by questionnaire scales: self-efficacy, social support for PA, and outcome expectancies. These variables were selected as the primary targets of the intervention. All scales were derived or modified from previously developed and validated scales (Table 2). The measurement properties of these scales are summarised in Table 2. The mean score of all relevant items was computed for each scale, for participants with a response rate of 75% or greater on the respective item [46]. Generally, internal consistency (Cronbach’s alpha) properties were satisfactory (Table 2). Information about barriers to PA was collected by asking the participants to; “Rate how relevant the listed barriers are for you”. The scale went from 0 (not a barrier) to 5 (very relevant).

**Table 2:** Measurement properties of psychosocial scales

**Variable**	**Number of items / response format**	**Example of sample items**	**Original reference source on which items were based**	**Cronbach`s alpha**
				**Baseline, FU1 and FU2 (range)**
*Social support*		Have your family/friends…	[47]	
- family	6 / 1 (never) - 5 (very often)	… Encouraged you to be physically active?		0.85-0.87
- friends	6 / 1 (never) - 5 (very often)			0.87-0.88
*Self-efficacy*	7 / 1 (not at all confident) - 7 (very confident)	I am confident I can participate in planned physical activity when… I am tired	[48]	0.87-0.89
*Outcome expectancies*	6 / 1 (unlikely) – 7 (very likely)	If I am regularly physically active in the next month… Iwill get in better shape		0.85-0.89

### Statistical analyses

The outcome data were analysed on a per protocol basis, without imputations. Delta PA scores and the potential psychosocial mediators were calculated (baseline to FU2), and used as the dependent variable in the analysis of covariance (ANCOVA), and with baseline measurements and age as covariates when calculating the significance of differences between the groups. Independent and paired-sample t tests were used to test differences between and within groups at the baseline, respectively, and t tests were used for the drop-out analysis. Effect sizes were calculated as: delta mean in the intervention group - delta mean in the control group / standard deviation of delta mean in the control group. According to Baron and Kenny (1986), several steps are required to demonstrate a mediation effect [49]. The criterion for a change in the hypothesised mediator to be associated with the change in PA was not met in the current study, and thus further analysis was not undertaken.

## Results

### Socio-demographics

Of the 150 participants at baseline, 124 (83%) were not born in Norway (first generation immigrants). The first generation immigrants had lived in Norway for an average of 20 years (range 1 to 38 years). One hundred and forty three were employed (95%), and 54% had college education. Most of the participants worked as either taxi drivers (48%) or white collar workers (31%). A high percentage of these men were overweight (93%) or obese (81%), were insulin resistant (73% scored > 2.5 in the homeostasis model assessment, developed by Matthews et al. [50]) or had the metabolic syndrome (50%), especially the taxi drivers [51]. Table 3 presents more of the baseline characteristics.

**Table 3:** Baseline characteristics for the intervention and the control group

**Characteristic**	**Intervention group**	**Control group**	**Mean difference**
	**(n = 89)**	**(n = 61)**	**(95% CI)**
Age (years)	35.7 (6.1)	39.7 (9.2)	−3.9 (−6.6 to −1.2)*
Weight (kg)	83.7 (12)	84.1 (14.4)	−0.3 (−4.7 to 4.1)
Height (cm)	174 (6.2)	174 (6.2)	0.6 (−1.3 to 2.7)
BMI (kg.m^-^²)	27.1 (3.2)	27.4 (4.2)	−0.2 (−1.5 to 0.9)
Waist circumference (cm)	98 (9)	99 (11)	−1.1 (−4.6 to 2.3)
Peak VO_2_ (ml·kg^-1^·min^-1^)†	33.9 (5.2)	34.7 (6.5)	−0.7 (−3.4 to 1.9)

Values are mean (standard deviation). The independent-sample *t* test was used to calculate significance of the difference between groups. *CI* confidence interval, *BMI* body mass index. * P = 0.005. † n = 30 and 69 for the control and the intervention groups, respectively.

### Attrition

Of the 133 participants who completed FU1, three declined the FU2 test, and 19 in the intervention group and 14 in the control group did not have valid accelerometer recordings at the FU2 test. At baseline, those with valid accelerometer recordings at FU2 (n = 97) had a lower postprandial glucose level (mean difference = − 1.2: 95% CI (confidence interval) = − 0.04 to - 2.4; P = 0.04) and fasting insulin level (mean difference = − 22: 95% CI = − 1.0 to - 43; P = 0.04) and higher CPM (mean difference = 49: 95% CI = 6.9 to 91; P = 0.02), compared with those with invalid accelerometer recordings at FU2 or drop-outs (n = 53).

### Physical activity

Table 4 displays the PA data at all the three measurements times for the intervention and control groups. The delta differences (baseline to FU2) in all PA variables differed significantly between groups. CPM on both weekends and workdays changed more in the intervention group than in the control group. The intervention group had 84 more minutes of MVPA and 7.7 fewer hours of inactive time per week than the control group.

**Table 4:** Mean and standard deviation of physical activity data at the three measurement times

	**Intervention group**			**Control group**					
	**Baseline**	**FU1**	**FU2**	**Baseline**	**FU1**	**FU2**	Adjusted Δ diff (95% CI)*	**Effect size**	**P-value**
Total PA level (CPM)	328 (138)	407 (149)	389 (137)	281 (118)	317 (129)	260 (99)	81 (36 to 126)	0.64	0.001
PA level on weekends (CPM)†	304 (150)	422 (188)	370 (150)	278 (142)	319 (147)	249 (136)	124 (44 to 203)	0.47	0.003
PA level on weekdays (CPM)	332 (143)	407 (157)	388 (148)	283 (128)	320 (144)	265 (98)	72 (23 to 120)	0.48	0.004
Sedentary time (hours.day^-1^)	8.4 (1.6)	7.9 (1.8)	7.7 (1.5)	8.9 (1.5)	8.9 (1.5)	9.3 (1.4)	−1.1 (−1.8 to −0.5)	−0.23	0.001
Light intensity PA (hours.day^-1^)	4.5 (1.4)	5.0 (1.2)	5.0 (1.2)	4.0 (1.0)	4.0 (1.1)	3.6 (1.0)	1.1 (0.6 to 1.6)	0.64	<0.001
MVPA (min.day^-1^)	35 (21)	46 (23)	44 (23)	28 (19)	33 (21)	27 (17)	12 (4.4 to 21.1)	0.72	0.003

* Difference (baseline to FU2), all variables were adjusted for their respective baseline value and age. ANCOVA was used to analyse the data. † n = 41 and 30 at the FU2 for the intervention and the control groups, respectively. *PA* physical activity, *CPM* counts per min, *MVPA* moderate and vigorous physical activity, *CI* confidence interval. *FU1* follow-up 1 (conducted immediately after the intervention), *FU2* follow-up 2 (conducted six months after the intervention).

The intervention group increased the total PA level from baseline to the FU2 by a mean of 36 CPM (95% CI = 4 to 70; P = 0.02), an increase of 10% (95% CI = 2 to 17) and time spent in MVPA by an average of 7.3 min∙day^-1^ (95% CI = 0.8 to 13.7; P = 0.03), an increase of 21% (95% CI = 10 to 31). The intervention group reduced sedentary time by a mean of 0.7 hours∙day^-1^ (95% CI = −0.3 to −1.1; P = 0.001), a reduction of 9% (95% CI = 1.5 to 16). The PA variables did not change in the control group.

In the intervention group, the only significant change in PA characteristics from FU1 to FU2 was sedentary time, which was lower at the FU2 test (mean difference −0.5, 95% CI = −0.04 to −0.9; P = 0.03). In the control group, CPM (mean difference −38, 95% CI = −64 to −11; P = 0.006) and light intensity (mean difference −0.5, 95% CI = −0.9 to −0.1; P = 0.01) decreased from FU1 to FU2.

### Social cognitive variables

Except for a higher score on outcome expectancies in the intervention group (mean difference = 0.6, 95% CI = 0.9 to 0.2; P < 0.01), none of the social cognitive variables differed between the two groups at baseline.

Support from family and outcome expectancies increased more from baseline to FU2 in the intervention group than in the control group (Table 5). In the intervention group the participants scored higher at FU2 on social support from family (mean difference = 0.3, 95% CI = 0.07 to 0.45; P = 0.008) and outcome expectancies (mean difference = 0.3, 95% CI = 0.08 to 0.6; P = 0.01). Self-efficacy and social support from friends did not change.

**Table 5:** Mean and standard deviation of social cognitive variables at all the three measurement times

	**Intervention group**			**Control group**					
	**Baseline**	**FU1**	**FU2**	**Baseline**	**FU1**	**FU2**	**Adjusted Δ diff**	**Effect size**	**P-value**
	**(n = 79-88)**	**(n = 71-74)**	**(n = 53-56)**	**(n = 54-58)**	**(n = 47-52)**	**(n = 37-39)**	(95% CI)*		
*Social support*									
- family	3.4 (0.8)	3.6 (0.8)	3.6 (0.9)	3.1 (0.8)	3.2 (0.7)	3.1 (0.6)	0.4 (0.1 to 0.7)	0.65	0.001
- friends	3.2 (0.9)	3.2 (0.9)	3.2 (0.8)	3.1 (0.9)	3.3 (0.8)	3.3 (0.8)	−0.1 (−0.4 to 0.1)	−0.12	0.4
*Self-efficacy*	4.1 (1.4)	4.1 (1.4)	4.1 (1.4)	3.8 (1.1)	3.9 (1.1)	3.5 (1.3)	0.5 (−0.1 to 1.1)	0.44	0.09
*Outcome expectancies*	6.3 (0.8)	6.4 (0.6)	6.5 (0.6)	5.7 (1.0)	5.7 (1.2)	5.7 (1.0)	0.7 (0.2 to 1.3)	0.38	0.01

* Difference (baseline to FU2), all variables were adjusted for their respective baseline value and age. ANCOVA was used to analyse the data. *CI* confidence interval, *FU1* follow-up 1 (conducted immediately after the intervention), *FU2* follow-up 2 (conducted six months after the intervention). Note; Self efficacy and outcome expectancies range from 1 to 7; Social support range from 1 to 5.

The intervention group perceived six of the 15 listed PA barriers to be less of an obstacle than did the control group. A significant change (from baseline to FU2) was found for the following barriers: time constraints (mean difference = 0.7, 95% CI = 0.0 to 1.5; P = 0.04), not the sporty type (mean difference = 1.2, 95% CI = 0.4 to 2.0; P = 0.002), lack of motivation (mean difference = 1.1, 95% CI = 0.3 to 2.0; P = 0.009), too expensive (mean difference = 1.0, 95% CI = 0.2 to 1.8; P = 0.01), don’t know how (mean difference = 1.2, 95% CI = 0.3 to 2.1; P = 0.008), and don’t find any activities that are okay to do (mean difference = 1.1, 95% CI = 0.3 to 2.0; P = 0.01).

Changes in the SCT constructs from baseline to FU1 did not correlate with changes in PA (CPM) from baseline to FU2 (data not shown). Therefore, mediation analysis could not be undertaken [49].

## Discussion

We have shown that a relatively simple PA programme can lead to both short- and long-term improvements in PA level among a sedentary, overweight, male, south Asian immigrant population, although the social cognitive mediators did not change markedly. The differences in CPM and MVPA between groups at FU1 were sustained and even increased at the six months follow-up (FU2). Inactive time, which did not change significantly from baseline to FU1, decreased significantly from baseline to FU2 in the intervention group compared with the control group.

Although the PA level increased from baseline to FU2, changes in the potential social cognitive mediators did not correlate with the change in PA, and mediation analyses could not be performed. The lack of correlation might be because there were other unmeasured factors that mediated the change in PA such as social support from the exercise leader or perceived access to facilities. Another possible explanation is that the intervention did not adequately address the potential mediators or that the intervention was not of sufficient length to achieve greater changes in these variables. It might also be that the social cognitive measures have not been validated on this group. Another explanation is a ceiling effect, meaning that the participants scored relatively high on many of the variables at baseline and further improvements were therefore difficult to achieve, and the small changes make it difficult to obtain significant correlations with PA. The results may also be biased because the participants were not physically active when the baseline testing was conducted and this may have made it difficult for them to answer the questions properly.

Compared with the control group, in the intervention group, only social support from family and outcome expectancies increased in the intervention group from baseline to FU2, although the change in self-efficacy was borderline significant. This means that the participants in the intervention group reported more support for being physically active, stronger beliefs that positive outcomes will follow participation in PA, and the perception that they had more control over being physically active when faced with barriers (e.g., time constraints). The intervention group perceived many of the PA barriers to be less of an obstacle for engaging in PA than did the control group. Although not individually correlated with changes in PA, the small differences in the aforementioned variables might together have contributed significantly to the increased engagement in PA. These changes could have resulted from specific programme strategies such as having access to low-threshold exercise classes with people who are similar to oneself (same level of physical fitness and skills), help in structuring the week and planning for PA, professionals being available to address PA related problems (trainer), and increased knowledge of PA.

Our results are encouraging because immigrants/ethnic minorities are considered an important group for health interventions but are also considered a challenging group to recruit into this kind of study. Some studies have addressed PA in other ethnic minority groups. In a review of 14 studies mainly on African-Americans, the interventions included a wide range of approaches: community oriented, family oriented, church based and home based [52]. Only four studies had a randomised controlled design. Overall, the results from the interventions were disappointing, and only two studies achieved changes in PA level. In the review by Taylor et al. (1998) the authors concluded that it is not clear which factors are critical for efficacious interventions but that community/participant involvement and a thorough assessment of needs, attitudes, preferences and unique barriers before the implementation of the intervention seem important [52]. Interventions should therefore be tailored specifically to the targeted ethnic group because ethnic minority populations might have specific barriers to and mediators of PA that differ from other groups [53]. The use of focus groups and people from the target group in the planning and implementation phases of the project in the PAMH study might therefore have been vital to the success of this programme. For example the focus groups meetings revealed a lack of knowledge of what PA is and that floor ball is a familiar sport; this information was useful for designing our intervention. In addition, members of the target group offered assistance in the recruitment phase and this may have been essential for the interest in the project that was created in the milieu. This view is supported by the findings in a systematic review in ethnic minorities, in which community participation was found to be very important for publicizing the intervention and increasing accessibility [37].

### Strengths and limitations

The PA programme used an SCT framework and was tailored to each individual’s specific interests and preferences, and aimed to enable the participants to incorporate more PA into their daily routine. The PA behaviour was targeted through multiple intervention components, but because we did not undertake any process evaluation, we do not know the contribution of each of the various components. The lack of anthropometric and blood measures preclude any conclusions about the clinical value of the increased PA level. However, the increased PA level (both CPM and MVPA) in the intervention group at FU1 was sustained at FU2, and one might expect that the improvements in waist circumference and insulin resistance demonstrated at FU1 would also be present at FU2. The intervention group had a significantly higher PA level than the control group at baseline. In theory, this could mean a lower potential for intervention, which might have led to underestimation of the long-term effects compared with a situation in which the two groups were similar at baseline. A major limitation is the attrition between baseline and FU2, which may have caused loss of the feature of randomisation [54]. Those who did drop out of the study had a lower PA level at baseline than those who did not drop out, and this might indicate that the intervention was more suitable for those who engaged in a minimum of PA at the start. It was not possible to satisfy the criterion of masking the exercise instructors or the participants from group allocation. RCTs that are not blinded tend to show greater intervention effects than RCTs that have this feature [55]. Finally, persons who respond to this type of study could be motivated to increase their PA level, and so the external validity regarding wider populations may be questionable. Internally, however, a randomised design should prevent this from affecting the results.

The major strengths of this follow up study include the randomised controlled design and repeated measurement of PA using objective PA data from accelerometers. Because there are no validated PA questionnaires for this group, the use of objective tools reduces the potential measurement error. Most accelerometers show good to very good correlations (r = 0.88) with energy expenditure during walking and running [43], and activity counts from the Actigraph accelerometer correlate well (r = 0.30 to 0.96) with PA energy expenditure measured using the doubly labeled water method [56]. However, accelerometers underestimate the energy cost of running > 9 km∙h^-1^, cycling, rowing and upper body movement [57], and do not capture water activities such as swimming. However, only a few participants in the current study reported engaging in swimming and cycling, and this limitation is therefore unlikely to have influenced the results.

## Conclusions

Our results show that a multi component PA programme can increase PA in a group of immigrant Pakistani men in both the short and long term. However, we do not known what factors mediated these changes in PA.

## Abbreviations

ANCOVA = Analysis of covariance; BMI = Body mass index; CI = Confidence interval; CPM = Counts per minute; CVD = Cardiovascular diseases; FU1 = Follow-up 1; FU2 = Follow-up 2; MVPA = Moderate- to vigorous intensity physical activity; PA = Physical Activity; PAMH = Physical activity and minority health; RCT = Randomised controlled trial; SCT = Social cognitive theory; T2D = Type 2 diabetes.

## Competing interest

The authors declare that there are no competing interests.

## Authors’ contributions

EA conceived and designed the study, performed all the statistical analysis, interpreted the data, and performed all testing and analysis. SAA conceived and designed the study. NWB interpreted the data. All authors participated in drafting and revising the article and approved the final version to be published.
